# CYP17A1 Maintains the Survival of Glioblastomas by Regulating SAR1-Mediated Endoplasmic Reticulum Health and Redox Homeostasis

**DOI:** 10.3390/cancers11091378

**Published:** 2019-09-16

**Authors:** Hong-Yi Lin, Chiung-Yuan Ko, Tzu-Jen Kao, Wen-Bin Yang, Yu-Ting Tsai, Jian-Ying Chuang, Siou-Lian Hu, Pei-Yu Yang, Wei-Lun Lo, Tsung-I Hsu

**Affiliations:** 1 Graduate Institute of Neural Regenerative Medicine, College of Medical Science and Technology, Taipei Medical University, Taipei 11031, Taiwan; d620105002@tmu.edu.tw (H.-Y.L.); ko680108@tmu.edu.tw (C.-Y.K.); geokao@tmu.edu.tw (T.-J.K.); chuangcy@tmu.edu.tw (J.-Y.C.); everywheresmile@gmail.com (S.-L.H.); peggy80123@hotmail.com (P.-Y.Y.); 12317@s.tmu.edu.tw (W.-L.L.); 2 Ph.D. Program for Neural Regenerative Medicine, College of Medical Science and Technology, Taipei Medical University and National Health Research Institutes, Taipei 11031, Taiwan; 3 TMU Research Center of Neuroscience, Taipei Medical University, Taipei 11031, Taiwan; 4 TMU Research Center of Cancer Translational Medicine, Taipei Medical University, Taipei 11031, Taiwan; 5 Graduate Institute of Medical Sciences, College of Medicine, Taipei Medical University, Taipei 11031, Taiwan; geniusbing@gmail.com (W.-B.Y.); terry1992@gmail.com (Y.-T.T.); 6 Division of Neurosurgery, Taipei Medical University-Shuang-Ho Hospital, New Taipei City 23561, Taiwan

**Keywords:** CYP17A1, glioblastoma, abiraterone, SAR1

## Abstract

Cytochrome P450 (CYP) 17A1 is an important steroidogenic enzyme harboring 17α-hydroxylase and performing 17,20 lyase activities in multiple steps of steroid hormone synthesis, including dehydroepiandrosterone (DHEA) biosynthesis. Previously, we showed that CYP17A1-mediated DHEA production clearly protects glioblastomas from temozolomide-induced apoptosis, leading to drug resistance. Herein, we attempt to clarify whether the inhibition of CYP17A1 has a tumor-suppressive effect, and to determine the steroidogenesis-independent functions of CYP17A1 in glioblastomas. Abiraterone, an inhibitor of CYP17A1, significantly inhibits the proliferation of A172, T98G, and PT#3 (the primary glioblastoma cells) by inducing apoptosis. In parallel, abiraterone potently suppresses tumor growth in mouse models through transplantation of PT#3 cells to the back or to the brain. Based on evidence that abiraterone induces endoplasmic reticulum (ER) stress, followed by the accumulation of reactive oxygen species (ROS), CYP17A1 is important for ER health and redox homeostasis. To confirm our hypothesis, we showed that CYP17A1 overexpression prevents the initiation of ER stress and attenuates ROS production by regulating SAR1a/b expression. Abiraterone dissociates SAR1a/b from ER-localized CYP17A1, and induces SAR1a/b ubiquitination, leading to degradation. Furthermore, SAR1 overexpression rescues abiraterone-induced apoptosis and impairs redox homeostasis. In addition to steroid hormone synthesis, CYP17A1 associates with SAR1a/b to regulate protein processing and maintain ER health in glioblastomas.

## 1. Introduction

Steroidogenesis-mediated neurosteroid production is important for the maintenance of brain health, including synapse formation, neural survival, and astrocyte proliferation [1,2,3,4]. Neurosteroids protect brain cells against stress, such as oxidative stress and glutamate cytotoxicity [5,6]. However, brain tumors are also protected by neurosteroids, attenuating the therapeutic effect of chemotherapy. Previously, we found that dehydroepiandrosterone (DHEA), a kind of neurosteroid, protects glioma cells from chemotherapy-induced apoptosis, leading to drug resistance [7,8]. In particular, the upregulation of DHEA in gliomas is caused by Sp1-mediated cytochrome P450 (CYP) 17A1 overexpression [7], implying that CYP17A1 is a potential target for glioma treatment. Hence, based on a previous study showing that CYP17A1 inhibition reduces DHEA production, we aimed to study whether the inhibition of CYP17A1 is effective at suppressing glioblastomas using abiraterone, which is a well-known chemotherapeutic drug for prostate cancer.

Interestingly, we clarify a steroidogenesis-independent function of CYP17A1 in this study. By regulating secretion-associated Ras-related GTPase (SAR) 1 expression, which is important for the process of protein trafficking from the ER to the Golgi apparatus [9], CYP17A1 is required for the survival of glioblastomas. The formation of COPII-coated vesicles, consisting of SEC13, SEC31, SEC23, and SEC24, by SAR1 is important for endoplasmic reticulum (ER) protein exportation, as it maintains the integrity of the ER [9]. The impairment of COPII functions and SAR1 deficiency causes the accumulation of unfolded proteins, leading to ER stress [10,11]. However, the role of SAR1-mediated COPII formation in tumor development has rarely been studied, and still remains unclear.

Herein, we clarify that COPII formation requires the interaction of CYP17A1 with SAR1a/b, and that inhibition of CYP17A1 by abiraterone abolishes this interaction, leading to ER stress. In addition, abiraterone obviously increases ROS production and impairs redox reactions, including the balance between reduced glutathione (GSH) and oxidized glutathione (GSSG), glutathione peroxidase (GPx), and glutathione reductase (GR) activities. In contrast, CYP17A1 overexpression prevents hydrogen peroxide-induced ER and oxidative stresses. Importantly, abiraterone exhibits a potent tumor-suppressive effect on glioblastomas in vitro and in vivo. A strategy targeting steroidogenesis, such as the inhibition of CYP17A1, is a potential option for glioblastoma treatment.

## 2. Results

### 2.1. Inhibition of CYP17A1 by Abiraterone Exhibits a Tumor-Suppressive Effect on Glioblastomas in Vitro and in Vivo

**Figure 1:**
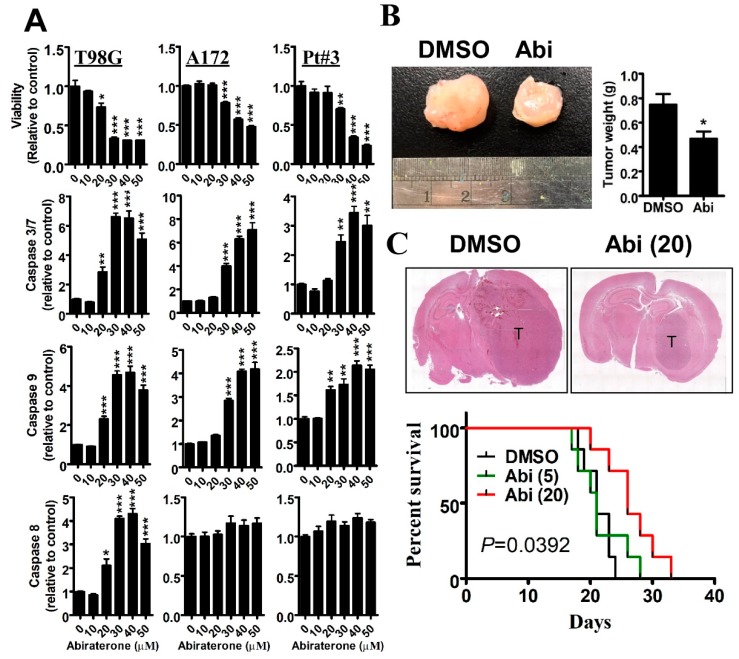
Effect of abiraterone (Abi) on glioblastomas in vitro and in vivo. (**A**) After treatment with different doses of Abi for 48 h, cells were subjected to MTT assay for evaluating viability. Cultured media were collected and analyzed for caspases 3/7, 8 and 9. Data were expressed as the relative value ± standard error of the mean (SEM) (* *p* < 0.05, ** *p* < 0.01 and *** *p* < 0.001 indicate the significant difference between the control group without treatment and other groups with Abi treatment) (**B**) Fourteen days after subcutaneous transplantation with PT#3 cells (1 × 10^6^), mice were administrated intravenously with 20 mg/kg Abi for 3 weeks (3 times/week). Excised tumors were photographed and weighed. Data were expressed as mean ± SEM (* *p* < 0.05). (**C**) Ten days after intracranial transplantation with PT#3 cells (2 × 10^5^), mice were administrated intravenously with 20 mg/kg Abi until death (3 times/week). After sacrificing, the brain was paraffin-embedded and subjected to slide preparation followed by hematoxylin and eosin (HE) staining. The date of death was recorded, and the survival rate was compared using the log-rank test.

Previously, we showed that CYP17A1 upregulation confers drug resistance to glioblastomas by increasing DHEA synthesis [7,8]. Hence, we aimed to study whether abiraterone, a well-known CYP17A1 inhibitor [12], potentially suppresses glioblastomas. As shown in Figure 1A, abiraterone significantly inhibited the survival of T98G, A172, and patient-derived PT#3 glioblastoma cells. Additionally, in a dose-dependent manner, abiraterone significantly induced apoptosis characterized by Caspase 3/7, 8, and 9 activities. Subsequently, we found that intravenous administration with abiraterone (20 mg/kg) significantly inhibited the growth of tumors, which were subcutaneously xenografted with PT#3 cells on the back (Figure 1B). Importantly, we established a mouse model that received intracranial transplantation of T98G cells, and found that abiraterone significantly suppressed tumor growth and extended the survival period (Figure 1C). These results indicate that the inhibition of CYP17A1 is an effective strategy for targeting glioblastomas.

### 2.2. Abiraterone Induces Endoplasmic Reticulum Stress and Reactive Oxygen Species Accumulation by Impairing Redox Reactions

**Figure 2:**
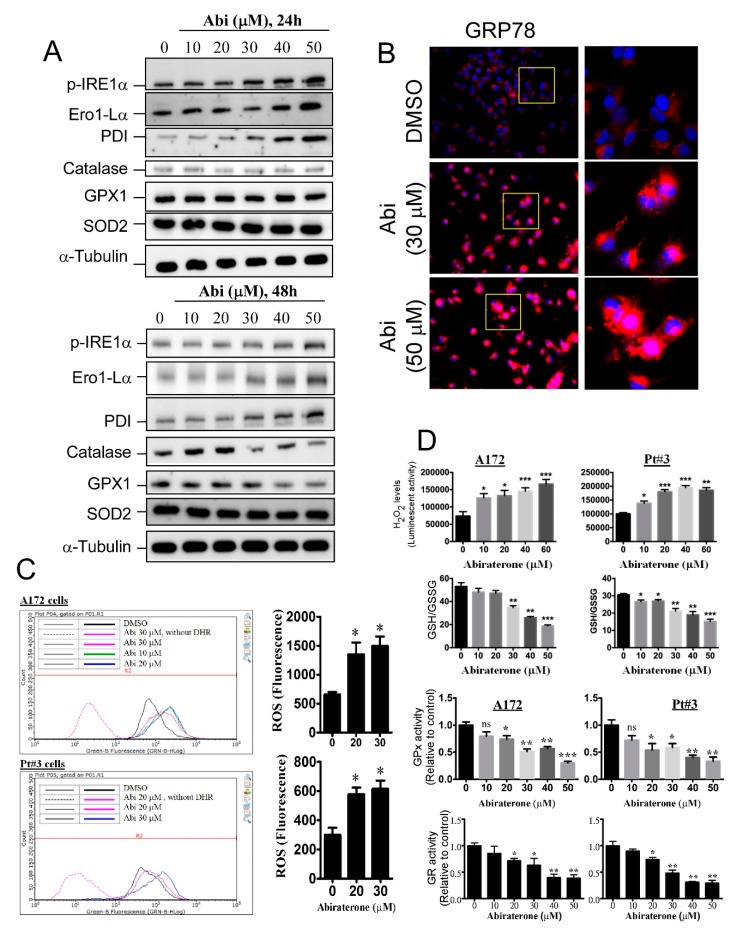
Abi induces endoplasmic reticulum (ER) stress and increases reactive oxygen species (ROS) production in glioblastomas. (**A**) After treatment with Abi, cell lysates were analyzed by western blotting using the indicated antibody. (**B**) After treatment for 24 h, cells were fixed, permeabilized, and stained using the anti-glucose-regulated protein (GRP) 78 antibody. (**C**) After treatment for 48 h, ROS levels in the cells were analyzed by dihydrorhodamine 123 (DHR) using flow cytometry. Data were expressed as mean ± SEM (* *p* < 0.05). (**D**) Effect of Abi on redox reactions. After 48 h of treatment, cells were harvested and analyzed for H_2_O_2_ levels, glutathione (GSH)/oxidized glutathione (GSSG) ratio, glutathione peroxidase (GPx) activity, and glutathione reductase (GR) activity. (* *p* < 0.05, ** *p* < 0.01, *** *p* < 0.001). None significance (ns) compared with control was indicated.

In addition to regulating steroid hormone metabolism, the CYP family is important for maintaining protein homeostasis and regulating detoxification in the ER [13,14]. We wanted to know whether CYP17A1 inhibition affects the ER, and we showed that the ER stress/unfolded protein response was obviously induced by abiraterone treatment for 24 h. As shown in Figure 2A, phosphorylated inositol-requiring 1α (p-IRE1α), ER oxidoreductin 1-Lα (Ero1-Lα), and protein disulphide isomerase (PDI), all of which are markers of ER stress, were obviously increased by abiraterone in a dose-dependent manner. In addition, abiraterone increased glucose-regulated protein (GRP) 78 expression, a classical characteristic of ER stress, further supporting the idea that CYP17A1 inhibition triggers ER stress (Figure 2B). Interestingly, proteins involved in ROS clearance, including catalase, glutathione peroxidase 1 (GPx1), and superoxide dismutase 2 (SOD2), were obviously decreased following abiraterone treatment for 48 h (Figure 2A). As confirmation that abiraterone affects redox homeostasis, leading to aberrant ROS production, we found that abiraterone significantly increased ROS and hydrogen peroxide levels in A172 and PT#3 cells (Figure 2C), accompanied by significant decreases in the GSH/GSSG ratio, GPx activity, and glutathione reductase (GR) activity (Figure 2D). This evidence indicates that CYP17A1 inhibition obviously initiates ROS accumulation and strong oxidative stress. Additionally, these results suggest that abiraterone-induced ER stress is followed by the dysregulation of redox reactions, leading to ROS accumulation and apoptosis.

### 2.3. CYP17A1 Prevents Reactive Oxygen Species Accumulation and Attenuates Reactive Oxygen Species-Induced Endoplasmic Reticulum Stress

**Figure 3:**
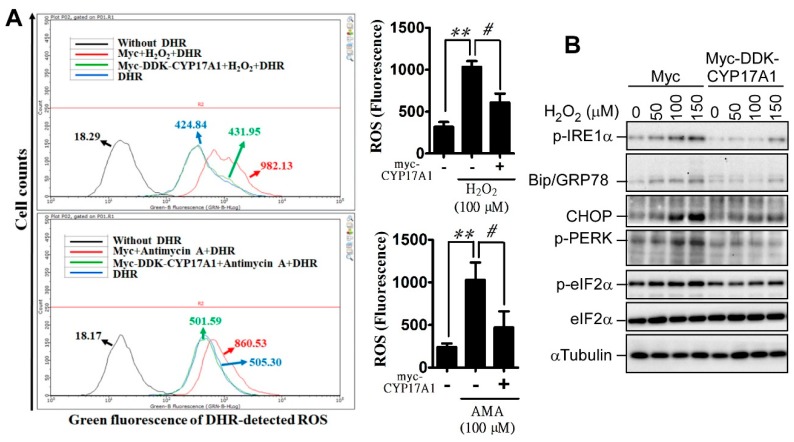

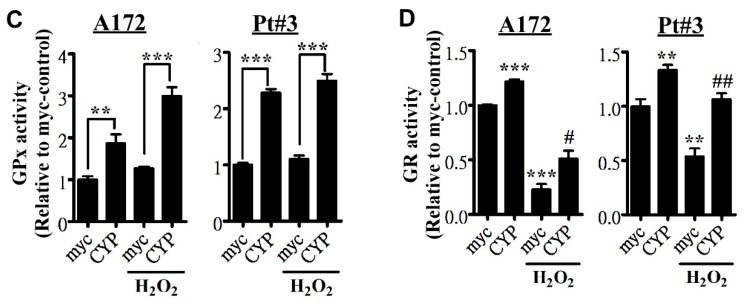
CYP17A1 attenuates hydrogen peroxide-induced ER and oxidative stresses. (**A**) After transfection with DDK (Flag)–Myc–CYP17A1 for 24 h, cells were treated with 100 μM H_2_O_2_ or with 50 μM antimycin a (AMA) for 24 h. Cells were harvested and mixed with DHR reagent for 30 min, followed by analysis using flow cytometry. Right panel: quantitative results. Data were expressed as mean ± SEM (^#^ *p* < 0.05, ** *p* < 0.01, # *p* < 0.05). (**B**) After treatment for 24 h, cell lysates were collected and analyzed by western blotting using the indicated antibody. (**C**,**D**) Cells with or without CYP17A1 overexpression were treated with 100 μM H_2_O_2_ for 24 h. Cells were harvested for analyzing GPx and GR activities. GPx results were expressed as a relative value ± SEM (** *p* < 0.01, *** *p* < 0.001). GR data were expressed as a relative value ± SEM (** *p* < 0.01 and *** *p* < 0.001 indicate the significant difference between myc-group without H_2_O_2_ and other groups; ^#^ *p* < 0.05, and ^##^ *p* < 0.01 indicate the significant difference between myc- and CYP17A1-expressed groups in the presence of H_2_O_2_ treatment).

To confirm the effect of CYP17A1 on redox homeostasis, we evaluated whether CYP17A1 has the potential to overcome oxidative stress induced by antimycin a (AMA) and hydrogen peroxide. Before studying the effect of CYP17A1, we confirmed that DDK (Flag)–Myc–CYP17A1 robustly increased the level of DHEA, indicating that the tagged CYP17A1 exhibits endogenous CYP17A1 activity (Supplementary Figure S1). Figure 3A shows that CYP17A1 overexpression significantly attenuated AMA- and hydrogen peroxide-induced ROS production. Additionally, hydrogen peroxide-induced ER stress, which is characterized by the presence of p-IRE1α, glucose-regulated protein (GRP) 78, CCAAT-enhancer-binding protein (C/EBP) homologous protein (CHOP), phosphorylated protein kinase R-like endoplasmic reticulum kinase (p-PERK), and p-eIF2α, was dramatically reduced by CYP17A1 (Figure 3B). Although we showed that CYP17A1 decreases ROS production, we still unable to exclude the involvement of DHEA, a major metabolite of CYP17A1. DHEA was shown to exhibit neuroprotective activity through preventing oxidative stress [15], and CYP17A1 overexpression also increased the level of DHEA (Supplementary Figure S1), leading to the decrease in ROS. Indeed, DHEA partially attenuated abiraterone-induced ROS production (Supplementary Figure S2), suggesting that CYP17A1-mediated antioxidant effect is partly through DHEA production. To estimate whether CYP17A1 affects redox reactions, we evaluated whether CYP17A1 regulates GPx and GR activities. As shown in Figure 3C,D, in the absence of hydrogen peroxide, CYP17A1 significantly upregulated the basal activities of GPx and GR. Moreover, CYP17A1 significantly rescued hydrogen peroxide-impaired GR activity, suggesting that CYP17A1 is an important redox regulator that prevents ROS accumulation.

### 2.4. CYP17A1 Regulates the Protein Stability of SAR1

**Figure 4:**
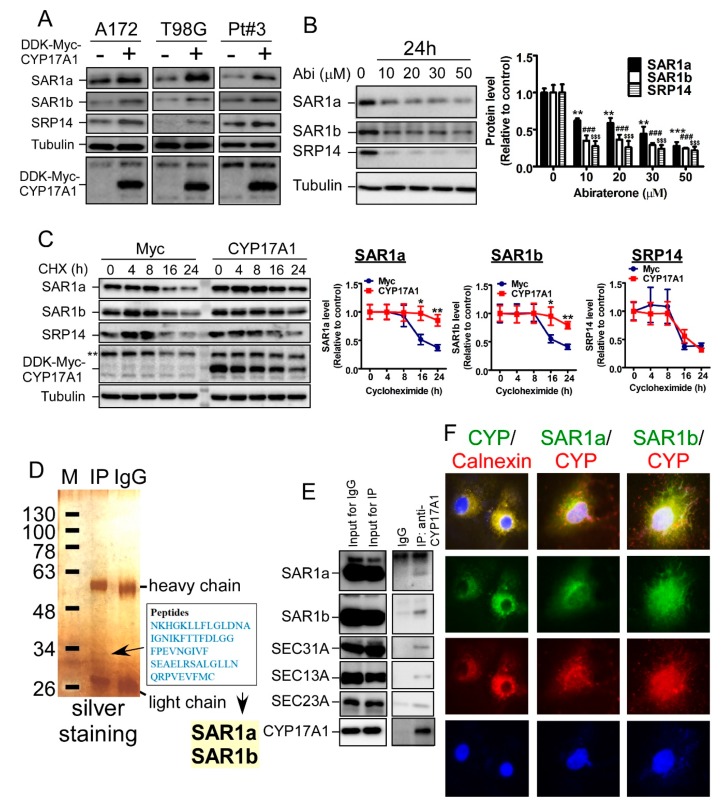
CYP17A1 interacts with secretion-associated Ras-related GTPase (SAR) 1a/b to regulate protein stability in ER. (**A**,**B**) Cells were analyzed by western blotting using antibodies targeting SAR1a, SAR1b, and SRP14. Quantitative results were expressed as a relative value ± SEM (** *p* < 0.01 and *** *p* < 0.001 indicate the significant difference in SAR1a protein expression between the control group without Abi treatment and other groups with Abi treatment; ^###^ *p* < 0.001 and ^$$$^ *p* < 0.001 indicate the significant difference in SAR1b and SRP14, respectively). (**C**) After transfection with DDK–Myc–CYP17A1, cells were treated with 20 μM cycloheximide (CHX) for indicated intervals. Cell lysates were analyzed by western blotting for SAR1a, SAR1b, and SRP14. Right panel: quantitative results. (* *p* < 0.05, ** *p* < 0.01). (**D**) Immune complex precipitated by the anti-CYP17A1 antibody was analyzed by silver staining. The arrow-indicated band was scooped out and transferred to proteomics analysis. Peptide sequences identified by LC-MS/MS were listed. (**E**) The immune complex was analyzed by western blotting, using the indicated antibodies. (**F**) After fixation and permeabilization, A172 cells were immunofluorescently stained using antibodies targeting CYP17A1 (CYP), SAR1a, SAR1b, and calnexin.

To investigate the mechanism underlying CYP17A1-mediated protection against oxidative stress, we attempted to identify proteins regulated by CYP17A1. To achieve this purpose, global protein expression of A172 with or without CYP17A1 overexpression was estimated by quantitative proteomics. In particular, proteomics and western blotting revealed that CYP17A1 increased the protein levels of secretion-associated Ras-related GTPase (SAR) 1a, SAR1b, and signal-recognition-particle (SRP) 14, all of which are ER-localized proteins and are important for protein processing (Supplementary Table S2 and Figure 4A). However, in contrast to SAR1 upregulation by CYP17A1 overexpression, DHEA was unable to increase SAR1 expression, indicating that SAR1 upregulation is caused by the CYP17A protein itself, not by DHEA (Supplementary Figure S3). On the other hand, abiraterone significantly decreased the expression of these three proteins (Figure 4B). Furthermore, in contrast to the lack of change in the mRNA level (Supplementary Figure S4), the protein half-lives of SAR1a and SAR1b were significantly decreased by abiraterone (Figure 4C). In addition, in the identification of CYP17A1-interacting proteins by proteomics, five peptides belonging to SAR1a and SAR1b were identified (Figure 4D), suggesting that CYP17A1 interacts with SAR1 to regulate protein stability. Because SAR1 is an important component of the COPII complex for protein trafficking to the Golgi apparatus, we confirmed that CYP17A1 associates with these components, including SAR1, SEC31A, SEC13A, and SEC23A (Figure 4E). Subsequently, we clarified that CYP17A1 co-localizes with calnexin, a marker of the ER, and co-localizes with SAR1a/b (Figure 4F), suggesting that ER-localized CYP17A1 regulates COPII formation for protein trafficking by interacting with SAR1a/b.

### 2.5. Inhibition of CYP17A1 Induces Oxidative Stress-Mediated Cell Death by Inducing SAR1 Ubiquitination

**Figure 5:**
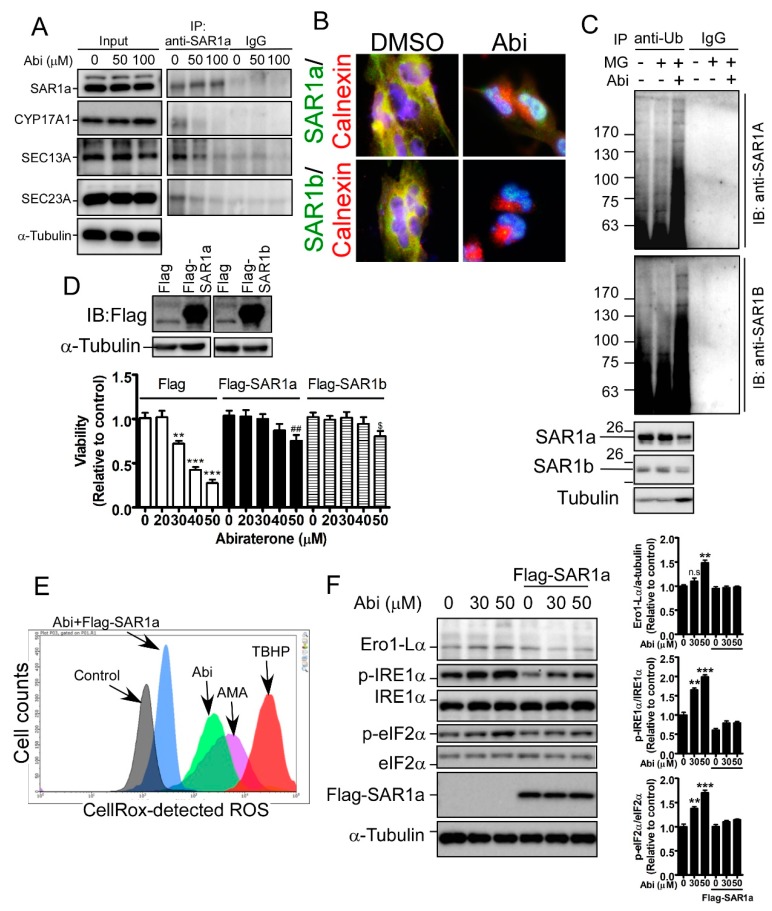
Abi induces ER and oxidative stresses by inducing SAR1a/b ubiquitination. (**A**) After treatment for 6 h, A172 cells were harvested and immuno-precipitated by the anti-SAR1a antibody. The complex was analyzed by western blotting. (**B**) A172 cells with or without Abi treatment for 6 h were stained by immunofluorescence, using antibodies targeting SAR1a, SAR1b, and calnexin. (**C**) After treatment with Abi for 24 h in the presence or absence of 20 μM MG132, A172 cells were subjected to immunoprecipitation and were analyzed by western blotting, using anti-ubiquitin, anti-SAR1a, and anti-SAR1b antibodies. (**D**) After transfection with Flag-SAR1a or Flag-SAR1b for 24 h, A172 cells were treated with Abi for 48 h. An MTT assay was performed to analyze viability. (** *p* < 0.01, *** *p* < 0.001 compared with Flag-transfected group without treatment; ^##^ *p* < 0.01 compared with Flag-SAR1a-transfected group without treatment; ^$^ *p* < 0.001 compared with Flag-SAR1b-transfected group without treatment). (**E**) A172 cells with or without Flag-SAR1a overexpression were treated with 50 μM Abi for 48 h, and ROS levels were determined by CellROX-mediated analysis. AMA and tert-butyl hydroperoxide (TBHP) treatments were positive controls for ROS accumulation. (**F**) A172 cells were treated with 50 μM Abi for 24 h; cell lysates were analyzed by western blotting. Right panel: quantitative results. (** *p* < 0.01, *** *p* < 0.001). None significance (n.s.) compared with the control group without Abi treatment was indicated.

Herein, we aim to elucidate how CYP17A1 regulates the protein stability of SAR1a, and confirmed that abiraterone obviously dissociates the interaction of CYP17A1 with SAR1a (Figure 5A). In addition, abiraterone dissociates SAR1a/b from ER-localized calnexin (Figure 5B), and induces SAR1a/b ubiquitination (Figure 5C). These results suggest that CYP17A1 is required for protein stabilization and ER localization of SAR1a/b. Subsequently, we found that overexpression of SAR1 significantly prevented abiraterone-induced cell death (Figure 5D), suggesting that the tumor-suppressive effect of CYP17A1 inhibition is mediated by the downregulation of SAR1a/b. Furthermore, SAR1a clearly prevented abiraterone-induced ROS production and ER stress (Supplementary Figure S5, Figure 5E,F), further supporting the idea that CYP17A1 maintains the health of the ER and regulates ROS homeostasis by regulating SAR1a/b protein stability.

## 3. Discussion

Previously, we clarified that CYP17A1-mediated DHEA synthesis is important for glioblastomas to develop drug resistance against temozolomide (TMZ) treatment [7,8]. Herein, we identified a novel function of CYP17A1 that is independent of steroidogenesis, and showed that CYP17A1 maintains the survival of glioblastomas by regulating SAR1a/b-mediated protein processing in the ER. In addition, CYP17A1 regulated redox reactions, including GPx and GR activities, preventing aberrant ROS accumulation and maintaining cellular health. Therefore, the inhibition of CYP17A1 by abiraterone exhibited an obvious tumor-suppressive effect on glioblastomas in vitro and in vivo. In particular, the abiraterone-impaired interaction of CYP17A1 with SAR1a/b initiated an unfolded protein response/ER stress, followed by the impairment of redox reactions, leading to ROS accumulation. Based on our evidence, CYP17A1 activity is important for SAR1 stabilization. Through maintaining SAR1 expression, CYP17A1 is capable of regulating protein trafficking. In the healthy condition for protein processing, ER stress is unable to be initiated, and thus ROS production is maintained at the low level. However, there is still a lack of direct evidence showing that CYP17A1 is involved in protein trafficking, although we found that CYP17A1 regulates SAR1a/b protein stability. Moreover, whether 17α-hydroxylase and 17,20 lyase activities of CYP17A1 are required to regulate SAR1 is still unclear. Thus, we are not sure whether SAR1 is a novel substrate of CYP17A1, even though we found an interaction of CYP17A1 with SAR1. Through 17α-hydroxylase and 17,20 lyase activities, multiple steps of steroidogenesis require CYP17A1, including the production of 17α-hydroxy-pregnenolone and DHEA [12,16]. In contrast, whether the enzymatic activity of CYP17A1 is involved in protein interactions, or whether it participates in other cellular processes, remains largely unclear. To understand the pathogenesis of diseases, including glioblastomas, caused by aberrant steroidogenesis, this mechanism requires study in the future.

Based on our data, CYP17A1 inhibition by abiraterone for 24 h induced ER stress/unfolded protein response, followed by robust accumulation of ROS at 48 h. Abiraterone increased Ero1-Lα expression, which is responsible for needed disulphide bond formation during ER stress; upregulated Ero1-Lα may synthesize mismatched disulfides, leading to an increase in hydrogen peroxide production [17]. Additionally, GSH is highly utilized to repair mismatched disulfides during ER stress, and depletion of GSH impairs redox reactions [17], leading to further upregulation of hydrogen peroxide. Hence, through activating Ero1-Lα and depleting GSH levels, CYP17A1 inhibition robustly induces ROS accumulation after ER stress.

The selectivity of abiraterone in targeting CYP17A1 remains arguable. In addition to the regulation of DHEA production by CYP17A1, abiraterone was shown to inhibit CYP21A1, which is responsible for the synthesis of deoxycortisosterone and deoxycortisol [18,19]. Moreover, several ER-localized CYPs harboring monooxygenase and epoxygenase activities were also found to be inhibited by abiraterone, such as CYP1A2, CYP2D6, CYP2C19, and CYP3A4, all of which are important for drug detoxification and the metabolism of arachidonate to epoxyeicosatrienoic acids [13,18,20,21,22]. Therefore, we cannot say definitively that abiraterone-induced ER stress and ROS accumulation are caused by CYP17A1 inhibition, based on our evidence. To improve the selectivity of inhibitory targeting of CYP17A1, orteronel (TAK-700), galaterone (TOK-001), and seviteronel (VT-464) were developed to treat diseases caused by the overproduction of androgens [12]. To specifically understand the role of CYP17A1 in glioblastomas, the effects of these inhibitors on glioblastomas need to be further elucidated.

In addition to regulating SAR1a/b expression, CYP17A1 also increased the expression of the SRP14 protein, which targets newly synthesized proteins by ribosomes on the ER membrane [23], without affecting mRNA expression and protein stability. This implies that the regulation of SRP14 by CYP17A1 is probably attributable to alterations in post-transcriptional modification and translational efficiency. Studies on CYP17A1 functions are still restricted in steroid hormone synthesis, and the role of ER-localized CYP17A1 in the ER remains uncovered. We propose that CYP17A1-mediated hydroxylation not only controls the synthesis of neurosteroids, but also regulates gene expression processes, from transcription to post-translational modification. Hydroxylation is important for regulating translational processes and protein stability [24,25]. 2-Oxoglutarate oxygenases are important ribosomal hydroxylases for protein synthesis [24], and Jumonji C domain-containing demethylase (JMJD) 4-mediated lysyl-hydroxylation of eRF1, a ribosome-associated protein, is essential for stop codon recognition by ribosomes for translation termination [26]. Proline hydroxylation mediated by prolyl hydroxylases has been shown to modulate the protein stability of HIF-1α under conditions of normoxia [27]. However, further work is required to elucidate the functional role of CYP17A1-catalyzed hydroxylation in tumor development.

Malignant brain tumors occur in males with higher prevalence (male/female ratio is 1.6:1) compared to the female population [28]. In addition, the female sex is also associated with longer survival and better response to therapy in glioblastoma [29,30]. However, the underlying mechanism still remains unclear, although cell cycle and integrin signaling were shown to dominate the difference in therapeutic response between male and female patients [28]. Particularly, sex differences in cell cycle and integrin signaling were consistently identified in young and post-menopausal adults, suggesting that circulating sex hormones from adrenal and gonadal glands are not involved in the differences. Interestingly, glioblastoma cells also synthesize and secret steroid hormones in the higher level than normal astrocytes, such as β-estradiol, DHEA, allopregnanolone, and 3-androstanediol (data not shown). Furthermore, in parallel with the upregulation of CYP17A1 [7], TMZ-resistant cells upregulate their steroidogenesis. CYP17A1 plays a central role in steroidogenesis, including the synthesis of androgens, estrogens, and corticosterone. A blockade of CYP17A1 activity dramatically decreases the production of both androgens and estrogens [31]. Therefore, we think that CYP17A1 is not involved in gender differences of glioblastoma. However, to further demonstrate whether CYP17A1-mediated steroidogenesis contributes to gender differences, the levels of multiple steroid hormones in both male and female glioblastomas will be globally estimated in the future.

## 4. Materials and Methods

### 4.1. Cell Lines, Chemical Compounds, and cDNA Clone

A172 and T98G cells were purchased from ATCC (Manassas, VA, USA), and all cell lines used in this study were cultured in Dulbecco's Modified Eagle Medium (DMEM) supplemented with 10% fetal bovine serum, as reported previously [7,8,32]. Abiraterone was purchased from Selleckchem (Houston, TX, USA) and dissolved in dimethyl sulfoxide (DMSO, MilliporeSigma Corporate, St. Louis, MO, USA). Hydrogen peroxide solution, antimycin a (AMA), and tert-butyl hydroperoxide (TBHP) were purchased from MilliporeSigma Corporate (St. Louis, MO, USA). DDK (Flag)–Myc–CYP17A1 was purchased from OriGene Technologies, Inc. (Rockville, MD, USA). Flag-SAR1a and -SAR1b constructs were purchased from GenScript^®^ (Piscataway, NJ, USA).

### 4.2. Primary Glioblastoma Cells

Human specimens were used with approval from the Institute Review Board/Ethics Committee (No. 201006011 and 201402018) from the office of human research at Taipei Medical University (Taipei, Taiwan), as described previously [8,32].

### 4.3. MTT (3-(4,5-dimethylthiazol-2-yl)-2,5-diphenyltetrazolium bromide) Assay

The protocol published in previous studies was followed [8,32].

### 4.4. Immunoprecipitation and Western Blotting

After collecting protein lysates in radioimmunoprecipitation assay (RIPA) buffer, 500 μg of protein was immunoprecipitated using 2 µg of the indicated antibody, followed by precipitation using 50 μL of protein A/G agarose (50% gel slurry in 20% ethanol). Immune complexes were analyzed by western blotting using the procedure described previously [7,8,32]. The primary antibodies used in this study are listed in Supplementary Table S1.

### 4.5. Immunofluorescence

Cells on the coverslips were fixed by 4% paraformaldehyde and permeabilized by 0.5% triton x-100. Subsequently, cells were stained using the anti-GRP78 antibody, followed by incubation with Alexa Fluor 578-conjugated anti-rabbit antibody in the presence of Hochest 33342. Fluorescent images were photographed with a fluorescence microscope (Leica, Wetzlar, Germany).

### 4.6. Caspase 3/7, 8, and 9 Detection

Caspase-Glo 3/7, 8, and 9 assay kits were purchased from Promega Inc. (Fitchburg, WI, USA), and used in accordance with the manufacturer’s instructions. Cultured media derived from T98G, A172, and PT#3 cells with or without abiraterone treatment were collected and mixed with assay reagents. A luminometer (Promega Inc.) was used to estimate caspase activities.

### 4.7. Reactive Oxygen Species (ROS) Analysis

Dihydrorhodamine 123 (DHR, Thermo Fisher Scientific, Waltham, MA, USA) and CellROX (Thermo Fisher Scientific) were used to measure ROS levels. After mixing with DHR or CellROX reagents for 30 min, fluorescence, representing ROS, was measured by flow cytometry and analyzed by GuavaSoft software (MerckMillipore, Bedford, MA, USA).

### 4.8. H_2_O_2_-Glo and Glutathione/Oxidized Glutathione-Glo Analysis, and Glutathione Peroxidase (GPx) and Glutathione Reductase (GR) Activity Analysis

H_2_O_2_-Glo (#G8820) and GSH/GSSG-Glo (#V6611) assay kits were purchased from Promega Inc. and used in accordance with the manufacturer’s instructions. The signal was estimated by GloMax Navigator (Promega Inc.). GPx (#K762) and GR (#K761) assay kits were purchased from Biovision Inc. (Milpitas, CA, USA). After the reaction, the signal was measured using an absorbance reader (Bio-Rad Laboratories, Inc., Hercules, CA, USA).

### 4.9. Proteomics

The proteomics experiments were assisted by Biotools Co., Ltd (New Taipei City, Taiwan)

### 4.10. Protein Sample Preparation and iTRAQ Labeling

Each specimen was taken out of the −80 °C freezer, and the protein amount in the cell lysate was determined using a BCA protein assay kit (Thermo Fisher Scientific). Two equal protein samples from each group (20 µg/sample) were subjected to reduction (5 mM tris-(2-carboxyethyl)-phosphine; MilliporeSigma Corporate), cysteine-blocking (10 mM methyl methanethiosulfonate; MilliporeSigma Corporate), and trypsin (1.6 µg, Promega) digestion at 37 °C for 16 h in solution containing 200 mM triethylammonium bicarbonate (TEABC). The peptides were then labeled with iTRAQ reagent (Applied Biosystems, Foster City, CA, USA), in accordance with the manufacturer’s protocol. After incubation at room temperature for 1 h, the four labeled peptide mixtures were pooled, dried by vacuum centrifugation, and stored at −80 °C until use.

### 4.11. In-Gel Digestion

The excised gel spot was first de-stained and then reduced with 10 mM dithiothreitol (DTT; MilliporeSigma Corporate, St. Louis, MO, USA) at 56 °C for 45 min, followed by cysteine-blocking with 55 mM iodoacetamide (IAM, MilliporeSigma Corporate, St. Louis, MO, USA) at 25 °C for 30 min. Samples were digested with sequencing-grade modified porcine trypsin (Promega, Madison, WI, USA) at 37 °C for 16 h. The peptides were then extracted from the gel, dried by vacuum centrifugation, and stored at −80 °C until use.

### 4.12. LC-MS/MS Analysis

The dried peptide mixtures (1.5 µg) were reconstituted in high-performance liquid chromatography (HPLC) buffer A (0.1% formic acid) and desalted using a homemade micro-column. The desalted peptides were loaded onto a reverse-phase column (Zorbax 300SB-C18, 0.3 × 5 mm; Agilent Technologies, Santa Clara, CA, USA) and separated with a reverse-phase column (HydroRP 2.5 µM, 75 μm inner diameter × 20 cm with a 15 μm tip) using a multi-step gradient of HPLC buffer B (99.9% acetonitrile/0.1% formic acid) for 150 min at a flow rate of 0.25 μL/min. The LC apparatus was coupled to a two-dimensional (2D) linear ion trap mass spectrometer (Orbitrap Elite; Thermo Fisher Scientific), operated using Xcalibur 2.2 software (Thermo Fisher, San Jose, CA, USA). The full-scan mass spectrometry (MS) was performed in the Orbitrap over a range of 400 to 1600 Da, and at a resolution of 120,000 at *m*/*z* 400. Internal calibration was performed using the ion signal of [Si(CH_3_)2O]6H+ at *m*/*z* 536.165365 as the lock mass. The 12 data-dependent MS/MS scan events (higher-energy collisional dissociation, HCD) were followed by one MS scan for the 12 most abundant precursor ions in the preview MS scan. The *m*/*z* values selected for MS/MS were dynamically excluded for 80 s, with a relative mass window of 15 ppm. The electrospray voltage was set to 2.0 kV, and the temperature of the capillary was set to 200 °C. MS and MS2 automatic gain control were set to 1000 ms (full scan) and 300 ms (MS2 for HCD), or 3 × 10^6^ ions (full scan) and 2 × 10^4^ ions (MS2 for HCD) for the maximum accumulated time or ions, respectively.

### 4.13. Protein Identification

The data analysis was carried out using Proteome Discoverer software (version 1.4, Thermo Fisher Scientific, Waltham, MA, USA). The MS/MS spectra were searched against the UniProt database (downloaded on March 16, 2016, extracted for *Homo sapiens*, 20,199 sequences) using the Mascot search engine (Matrix Science, London, UK; version 2.5). For peptide identification, a mass tolerance of 10 ppm for intact peptide masses and 0.05 Da for HCD fragment ions was permitted, with allowance for two missed cleavages from the trypsin digestion. Oxidized methionine, acetyl (protein N-terminal), tandem mass tag (TMT, N-terminal), and TMT (lysine) were used as variable modifications. Methylthio (cysteine) was used as the fixed modification. Peptide-spectrum matches (PSMs) were then filtered based on high confidence and a Mascot search engine rank 1 for peptide identification, to ensure that the overall false discovery rate was below 0.01. With the exception of phosphopeptides, proteins with single peptide hits were removed.

### 4.14. Tumor Transplantation

Animal experiments were approved by the Institutional Animal Care and Use Committee of Taipei Medical University. PT#3 cells were subcutaneously xenografted onto the back or were intracranially transplanted into 8-week-old NOD.CB17-Prkdc^scid^/NcrCrl (NOD/SCID) male mice. For subcutaneous transplantation, 1 × 10^6^ cells in 50 μL DMEM were prepared. Fourteen days after transplantation, mice were administrated intravenously with abiraterone (three times/week) for an additional three weeks. Tumors were excised and weighted.

For intracranial transplantation, 2 × 10^5^ cells in 5 μL DMEM were prepared and injected at a depth of 3 mm using stereotactic guidance (RWD Life Science Inc., San Diego, CA, USA) and a microprocessor single syringe (Harvard Apparatus, Holliston, MA, USA). Fourteen days after transplantation, mice were administrated intravenously with abiraterone (three times/week) until the death of the mice. The dates of death were recorded, and mice were analyzed with a Kaplan–Meier plot followed by the Log–Rank test to uncover significant differences. Ten micrometers of paraffin-embedded brain slices were prepared and stained by hematoxylin and eosin.

### 4.15. Statistical Analysis

The comparison between two groups was performed using Student’s *t*-test. A *p*-value < 0.05 was considered to represent a significant difference.

## 5. Conclusions

Based on the current and previous findings, blockade of CYP17A1 activity is a potential strategy to suppress glioblastomas. Abiraterone effectively attenuates the upregulation of DHEA production in glioblastomas, and obviously impairs SAR1-mediated protein processing, leading to the initiation of ER stress, ROS accumulation, and apoptosis. Therapeutic strategies targeting steroidogenesis in endocrine-related cancers may be a new medical option considered for glioblastoma treatment.

## Supplementary Materials

The following are available online at https://www.mdpi.com/2072-6694/11/9/1378/s1, Figure S1: The effect of DDK-CYP17A1 overexpression on DHEA production, Figure S2: DHEA attenuates abiraterone-induced ROS production, Figure S3: Effect of DHEA on SAR1a/b expression, Figure S4: Effect of abiraterone (A) on mRNA levels of SAR1a/b and SRP14, Supplementary Figure S5: Effect of SAR1a on Abi-induced ROS production, Table S1: The antibody list, Table S2: Upregulated proteins by CYP17A1 overexpression.
