# Supplementary material for: CYP17A1 Maintains the Survival of Glioblastomas by Regulating SAR1-Mediated Endoplasmic Reticulum Health and Redox Homeostasis
Source: Cancers (Basel). 2019 Sep 16;11(9):1378. doi: 10.3390/cancers11091378 (PMC6770831; doi:10.3390/cancers11091378)
Supplement: Supplementary file 1 [file cancers-11-01378-s001.pdf]

Supplementary Materials:

CYP17A1 Maintains the Survival of Glioblastomas by Regulating SAR1-Mediated Endoplasmic Reticulum Health and Redox Homeostasis

Hong-Yi Lin, Chiung-Yuan Ko, Tzu-Jen Kao, Wen-Bin Yang, Yu-Ting Tsai, Jian-Ying Chuang, Siou-Lian Hu, Pei-Yu Yang, Wei-Lun Lo and Tsung-I Hsu

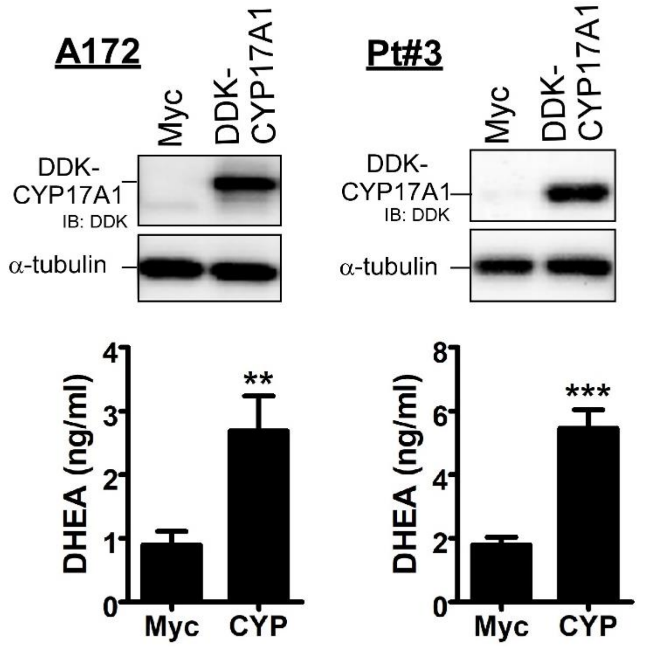

Figure S1. The effect of DDK-CYP17A1 overexpression on DHEA production. After transfection with the indicated expression plasmid for 24 h, cellular medium was collected for analysis by ELISA targeting DHEA.

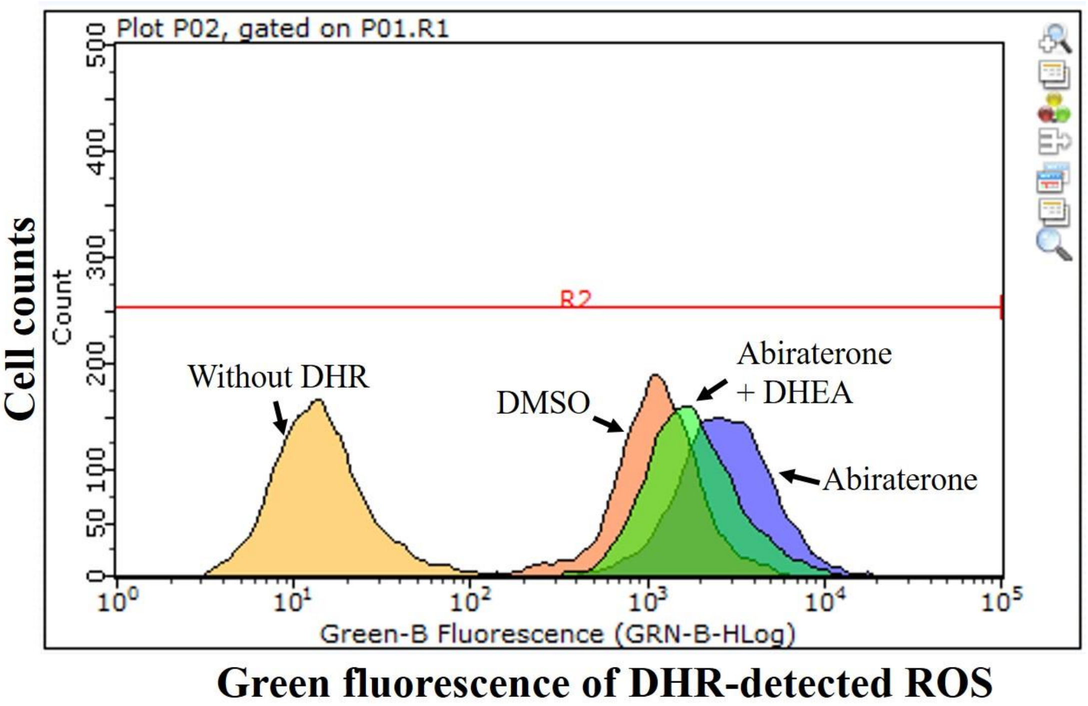

Figure S2. DHEA attenuates abiraterone-induced ROS production. A172 cells were treated with 50 μM abiraterone in the presence of 10 μM DHEA for 48 h, and cells were stained by DHR for ROS estimation using flow cytometry.

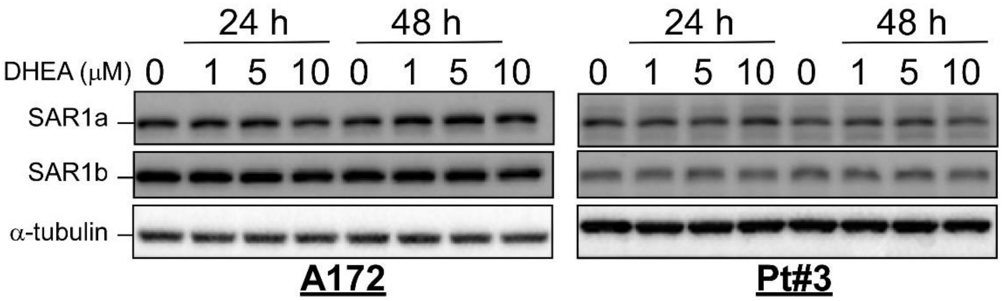

Figure S3. Effect of DHEA on SAR1a/b expression. After treatment with DHEA, cell extracts were collected and subjected to western blotting using the anti-SAR1a or anti-SAR1b antibody.

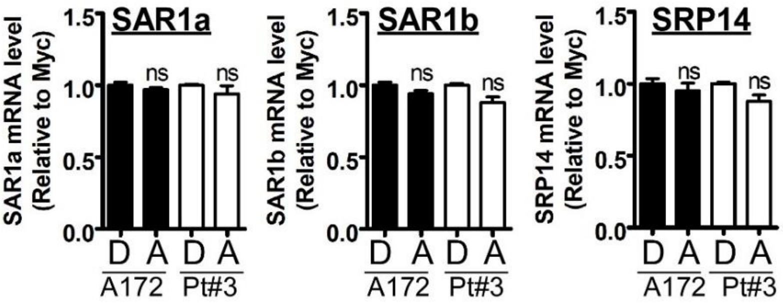

Figure S4. Effect of abiraterone (**A**) on mRNA levels of SAR1a/b and SRP14. After treatment with DMSO (**D**) or 50 μM abiraterone (**A**) for 24 h, total RNA was extracted and reversely transcripted to cDNA followed by real time qPCR using indicated primers. The significant difference between D- and A-groups was analyzed by Student's *t* test. "ns" means "not significant".

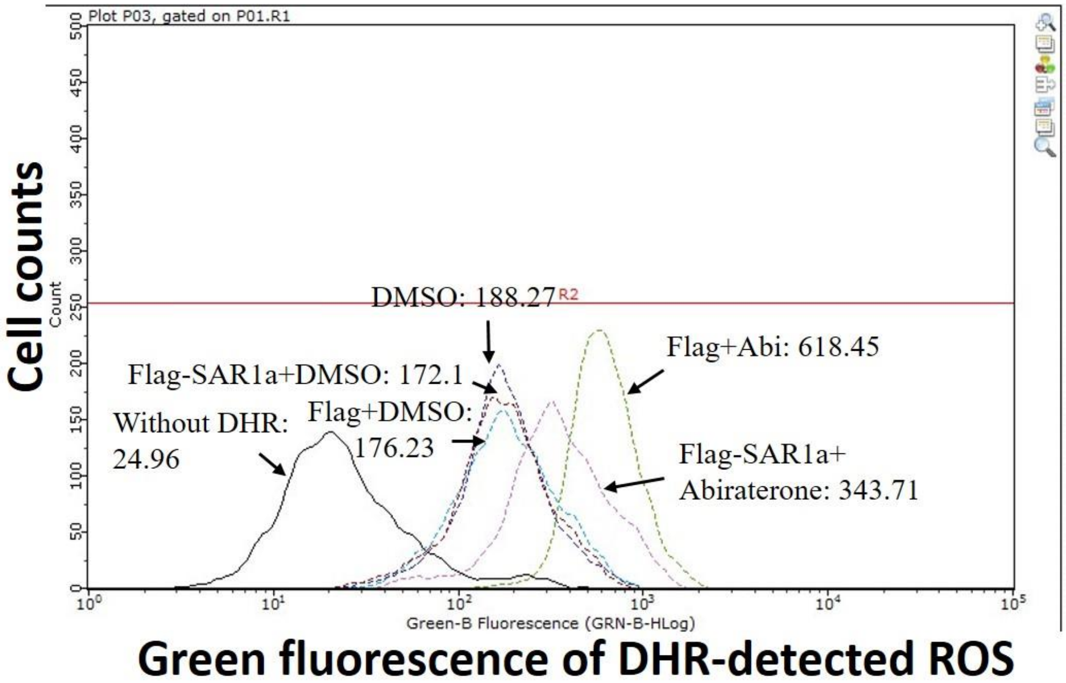

Figure S5. Effect of SAR1a on Abi-induced ROS production. After transfection with Flag or Flag-SAR1a plasmid for 24 h, Pt#3 cells were treated with Abi for 48 h. Subsequently, cells were harvested and stained by DHR for ROS analysis using flow cytometry. Cell counts representing ROS-positive cells were indicated.

Table S1. The antibody list.

Antibody	Brand	Titer
CYP17A1	abcam (Cambridge, UK)	WB: 1:1000<br>IF: 1:100
◎-tubulin	MilliporeSigma Corporate (St. Louis, MO, USA)	WB: 1:15000
Flag-tag	MilliporeSigma Corporate	WB: 1:5000
Ubiquitin	GeneTex International Corporation (HsinChu, Taiwan)	WB: 1:3000
p-eIF2α	Cell Signaling Technology (Danvers, MA, USA)	WB: 1:1000
eIF2α	Cell Signaling Technology	WB: 1:1000
p-IRE1α	abcam	WB: 1:1000
CHOP	Cell Signaling Technology	WB: 1:500
DDK-Myc	Origene (Rockville, MD, USA)	WB: 1:3000
Ero1-Lα	Cell Signaling Technology	WB: 1:1000
Catalase	Cell Signaling Technology	WB: 1:1000
SOD2	GeneTex International Corporation	WB: 1:1000
GRP78	GeneTex International Corporation	WB: 1:5000<br>IF: 1:300
p-PERK	Cell Signaling Technology	WB: 1:1000
Calnexin	Cell Signaling Technology	IF: 1:200
GPx1	GeneTex International Corporation	WB: 1:1000
SAR1a	GeneTex International Corporation	WB: 1:1000<br>IF: 1:100
SAR1b	GeneTex International Corporation	WB: 1:1000<br>IF: 1:100
SRP14	GeneTex International Corporation	WB: 1:1000<br>IF: 1:100
SEC31A	ABclonal (Manhattan Beach, CA, USA)	WB: 1:1000
SEC13A	ABclonal	WB: 1:1000
SEC23A	ABclonal	WB: 1:1000

Table S2. Upregulated proteins by CYP17A1 overexpression. SAR1a, SAR1b and SRP14 were further analyzed. Cells transfected with DDK-Myc-CYP17A1 or Myc for 24h, A172 cells were harvested for proteomics analysis.

Accession	Description	Gene symbol	Σ# PSMs	CYP17A1/Myc		
				Ratio	Count	Variability [%]
Q9NR31	GTP-binding protein SAR1a OS=Homo sapiens GN=SAR1A PE=1 SV=1 - [SAR1A_HUMAN]	SAR1A	4	1.495	3	1.8
Q9Y6B6	GTP-binding protein SAR1b OS=Homo sapiens GN=SAR1B PE=1 SV=1 - [SAR1B_HUMAN]	SAR1B	3	1.371	2	18.0
P37108	Signal recognition particle 14 kDa protein OS=Homo sapiens GN=SRP14 PE=1 SV=2 - [SRP14_HUMAN]	SRP14	3	1.371	3	0.6
P98179	RNA-binding protein 3 OS=Homo sapiens GN=RBM3 PE=1 SV=1 - [RBM3_HUMAN]	RBM3	2	1.330	2	33.7
P17858	ATP-dependent 6-phosphofructokinase, liver type OS=Homo sapiens GN=PFKL PE=1 SV=6 - [PFKAL_HUMAN]	PFKL	5	1.291	1	
P99999	Cytochrome c OS=Homo sapiens GN=CYCS PE=1 SV=2 - [CYC_HUMAN]	CYCS	7	1.267	7	21.5
P48556	26S proteasome non-ATPase regulatory subunit 8 OS=Homo sapiens GN=PSMD8 PE=1 SV=2 - [PSMD8_HUMAN]	PSMD8	2	1.242	2	37.0
Q13526	Peptidyl-prolyl cis-trans isomerase NIMA-interacting 1 OS=Homo sapiens GN=PIN1 PE=1 SV=1 - [PIN1_HUMAN]	PIN1	2	1.235	2	26.1
O95881	Thioredoxin domain-containing protein 12 OS=Homo sapiens GN=TXNDC12 PE=1 SV=1 - [TXD12_HUMAN]	TXNDC12	2	1.207	2	39.9
O95336	6-phosphogluconolactonase OS=Homo sapiens GN=PGLS PE=1 SV=2 - [6PGL_HUMAN]	PGLS	6	1.190	5	18.1
Q68CZ2	Tensin-3 OS=Homo sapiens GN=TNS3 PE=1 SV=2 - [TENS3_HUMAN]	TNS3	2	1.185	1	
O15067	Phosphoribosylformylglycinamidine synthase OS=Homo sapiens GN=PFAS PE=1 SV=4 - [PUR4_HUMAN]	PFAS	3	1.184	3	14.1
O75822	Eukaryotic translation initiation factor 3 subunit J OS=Homo sapiens GN=EIF3J PE=1 SV=2 - [EIF3J_HUMAN]	EIF3J	5	1.183	3	4.0
Q9P1F3	Costars family protein ABRACL OS=Homo sapiens GN=ABRACL PE=1 SV=1 - [ABRAL_HUMAN]	ABRACL	2	1.180	1	
Q15019	Septin-2 OS=Homo sapiens GN=SEPT2 PE=1 SV=1 - [SEPT2_HUMAN]	SEPT2	8	1.173	7	23.7
P78527	DNA-dependent protein kinase catalytic subunit OS=Homo sapiens GN=PRKDC PE=1 SV=3 - [PRKDC_HUMAN]	PRKDC	2	1.162	2	31.3
P00918	Carbonic anhydrase 2 OS=Homo sapiens GN=CA2 PE=1 SV=2 - [CAH2_HUMAN]	CA2	3	1.157	3	23.9
O15372	Eukaryotic translation initiation factor 3 subunit H OS=Homo sapiens GN=EIF3H PE=1 SV=1 - [EIF3H_HUMAN]	EIF3H	2	1.152	2	19.3
Q96FQ6	Protein S100-A16 OS=Homo sapiens GN=S100A16 PE=1 SV=1 - [S10AG_HUMAN]	S100A16	3	1.151	3	7.2
P07602	Prosaposin OS=Homo sapiens GN=PSAP PE=1 SV=2 - [SAP_HUMAN]	PSAP	7	1.150	7	24.6
P28072	Proteasome subunit beta type-6 OS=Homo sapiens GN=PSMB6 PE=1 SV=4 - [PSB6_HUMAN]	PSMB6	3	1.146	3	8.7

Q16643	Drebrin OS=Homo sapiens GN=DBN1 PE=1 SV=4 - [DREB_HUMAN]	DBN1	2	1.143	2	7.0
P33991	DNA replication licensing factor MCM4 OS=Homo sapiens GN=MCM4 PE=1 SV=5 - [MCM4_HUMAN]	MCM4	8	1.141	8	25.0
P13798	Acylamino-acid-releasing enzyme OS=Homo sapiens GN=APEH PE=1 SV=4 - [ACPH_HUMAN]	APEH	5	1.140	5	20.1
Q96HC4	PDZ and LIM domain protein 5 OS=Homo sapiens GN=PDLIM5 PE=1 SV=5 - [PDLI5_HUMAN]	PDLIM5	2	1.139	2	18.1
P63167	Dynein light chain 1, cytoplasmic OS=Homo sapiens GN=DYNLL1 PE=1 SV=1 - [DYL1_HUMAN]	DYNLL1	4	1.139	3	40.8
Q9NUQ9	Protein FAM49B OS=Homo sapiens GN=FAM49B PE=1 SV=1 - [FA49B_HUMAN]	FAM49B	3	1.138	3	0.8
Q9UK76	Hematological and neurological expressed 1 protein OS=Homo sapiens GN=HN1 PE=1 SV=3 - [HN1_HUMAN]	HN1	4	1.135	4	31.1
P61923	Coatomer subunit zeta-1 OS=Homo sapiens GN=COPZ1 PE=1 SV=1 - [COPZ1_HUMAN]	COPZ1	2	1.134	2	70.9
P78344	Eukaryotic translation initiation factor 4 gamma 2 OS=Homo sapiens GN=EIF4G2 PE=1 SV=1 - [IF4G2_HUMAN]	EIF4G2	2	1.132	1	
P62820	Ras-related protein Rab-1A OS=Homo sapiens GN=RAB1A PE=1 SV=3 - [RAB1A_HUMAN]	RAB1A	8	1.130	2	3.2
Q92820	Gamma-glutamyl hydrolase OS=Homo sapiens GN=GGH PE=1 SV=2 - [GGH_HUMAN]	GGH	3	1.127	3	5.0
Q13162	Peroxiredoxin-4 OS=Homo sapiens GN=PRDX4 PE=1 SV=1 - [PRDX4_HUMAN]	PRDX4	4	1.126	2	11.0
P26368	Splicing factor U2AF 65 kDa subunit OS=Homo sapiens GN=U2AF2 PE=1 SV=4 - [U2AF2_HUMAN]	U2AF2	3	1.124	3	2.3
P08670	Vimentin OS=Homo sapiens GN=VIM PE=1 SV=4 - [VIME_HUMAN]	VIM	65	1.124	63	12.9
P84098	60S ribosomal protein L19 OS=Homo sapiens GN=RPL19 PE=1 SV=1 - [RL19_HUMAN]	RPL19	2	1.124	2	0.2
Q14247	Src substrate cortactin OS=Homo sapiens GN=CTTN PE=1 SV=2 - [SRC8_HUMAN]	CTTN	11	1.123	11	6.0
Q13509	Tubulin beta-3 chain OS=Homo sapiens GN=TUBB3 PE=1 SV=2 - [TBB3_HUMAN]	TUBB3	38	1.122	3	21.5
Q9P2E9	Ribosome-binding protein 1 OS=Homo sapiens GN=RRBP1 PE=1 SV=4 - [RRBP1_HUMAN]	RRBP1	3	1.121	3	6.6
